# Mechanical characterization of isolated mitochondria under conditions of oxidative stress

**DOI:** 10.1063/5.0111581

**Published:** 2022-11-17

**Authors:** Yesaswini Komaragiri, Muzaffar H. Panhwar, Bob Fregin, Gayatri Jagirdar, Carmen Wolke, Stefanie Spiegler, Oliver Otto

**Affiliations:** 1 Zentrum für Innovationskompetenz: Humorale Immunreaktionen bei kardiovaskulären Erkrankungen, Universität Greifswald, Friedrich-Ludwig-Jahn-Str. 15a, 17489 Greifswald, Germany; 2 Deutsches Zentrum für Herz-Kreislauf-Forschung e.V., Standort Greifswald, Universitätsmedizin Greifswald, Fleischmannstr. 42, 17489 Greifswald, Germany; 3 Institut für Physik, Universität Greifswald, Felix-Hausdorff-Strasse 6, 17489 Greifswald, Germany; 4 Institut für Medizinische Biochemie und Molekularbiologie, Universitätsmedizin Greifswald, Ferdinand-Sauerbruch-Strasse, 17475 Greifswald, Germany

## Abstract

Mechanical properties have been proven to be a pivotal parameter to enhance our understanding of living systems. While research during the last decades focused on cells and tissues, little is known about the role of organelle mechanics in cell function. Here, mitochondria are of specific interest due to their involvement in numerous physiological and pathological processes, e.g., in the production and homeostasis of reactive oxygen species (ROS). Using real-time fluorescence and deformability cytometry, we present a microfluidic technology that is capable to determine the mechanical properties of individual mitochondria at a throughput exceeding 100 organelles per second. Our data on several thousands of viable mitochondria isolated from rat C6 glial cells yield a homogenous population with a median deformation that scales with the applied hydrodynamic stress. In two proof-of-principle studies, we investigated the impact of exogenously and endogenously produced ROS on mitochondria mechanics. Exposing C6 cells to hydrogen peroxide (H_2_O_2_) triggers superoxide production and leads to a reduction in mitochondria size while deformation is increased. In a second study, we focused on the knockout of *tafazzin*, which has been associated with impaired remodeling of the mitochondrial membrane and elevated levels of ROS. Interestingly, our results reveal the same mechanical alterations as observed after the exposure to H_2_O_2_, which points to a unified biophysical mechanism of how mitochondria respond to the presence of oxidative stress. In summary, we introduce high-throughput mechanical phenotyping into the field of organelle biology with potential applications for understanding sub-cellular dynamics that have not been accessible before.

## INTRODUCTION

Mitochondria are fundamental cell organelles involved in energy supply by ATP production through oxidative phosphorylation that powers cellular metabolism.^1^ Due to their highly dynamic nature, mitochondria are responsible for the coordination of many cellular processes, including the synthesis of phospholipids, calcium homeostasis, production, and maintenance of reactive oxygen species (ROS), as well as apoptosis, which are all essential for the development of an organism.^2,3^ Structurally, mitochondria are rod-shaped organelles consisting of two functionally distinct membranes separated by the inner membrane space and matrix.^4,5^

Cellular organelles like mitochondria account for more than half of the cytoplasm's volume, making them essential for mechanotransduction^6^ and mediating internal and external physical forces in the vicinity of the plasma membrane.^7,8^ Mitochondrial motion, fission, and fusion are frequently accompanied by dynamic shape changes and corresponding deformations, suggesting that mechanical properties play an essential role in these processes.^9–11^ For example, in neuronal cells, mitochondria undergo deformations within narrow axonal spaces to reach the energy requirement for ion exchange at active synapses.^12,13^ It has been shown that the application of mechanical stress leads to morphological changes, i.e., the elongation of mitochondria, followed by the release of excess cytochrome *c*.^10,11^ The unabated increase in cytochrome *c*, in turn, causes a decline in the mitochondrial membrane potential, increases ROS production, and ultimately triggers apoptosis as well as cell death.^7–15^

Cell and tissue mechanics have been intensively studied in the context of fundamental research but also from a translational perspective like disease diagnosis or drug screening.^16–25^ With the exception of mitochondria and the cell nucleus, the role of organelle mechanics in cell physiology or pathology is mostly unexplored.^26^ The nucleus is known to undergo complex changes in position, shape, and polarity.^27^ When cells are subjected to physical stress, the nuclear envelope, particularly the nuclear lamina, shields the nuclear interior. Any mutations in the nuclear protein lamin^28^ have been shown to increase nucleus stiffness, leading to disorders, such as Emery–Dreifuss muscular dystrophy (EDMD), dilated cardiomyopathy, familial partial lipodystrophy (FPLD), and the premature aging disease Hutchinson–Gilford progeria syndrome.^29,30^

To date, mitochondria mechanics and their relevance for biological function have already been studied by atomic force microscopy (AFM).^11^ During a myocardial infarction, mitochondria swell, causing alterations in their outer membrane, which are accompanied by stiffness changes. Furthermore, a micropipette aspiration study revealed that differences in physicochemical parameters such as osmotic pressure or pH had a substantial effect on mitochondrial membrane deformability.^31^ Wang *et al.* showed that low osmolarity results in reduced mitochondrial stiffness while deformation was also pH dependent. Though both methods already provided important insights into fundamental aspects of mitochondria mechanics, their relatively low throughput and the necessity to perform measurements in the presence of surface contacts, render applications that require large sample sizes of viable organelles challenging.^19–22^

The ability to mechanically characterize mitochondria in suspension would be of specific importance to understand their function. For example, fission and fusion are known to play a role in cellular quality control, in the generation of new mitochondria and in the cellular response to oxidative stress.^32^ While mitochondrial fission and fusion have been thoroughly investigated on a molecular level,^33^ little is known about the impact of material properties on these dynamic processes. Interestingly, theoretical models predict that alterations in the Gaussian curvature modulus might lead to an energy barrier impeding organelle fission and fusion.^34^

Here, we explored the possibility to apply real-time fluorescence and deformability cytometry (RT-FDC) to characterize the mechanical properties of individual mitochondria in flow. As a model system, we used rat C6 glial cells in the absence and presence of oxidative stress to interfere with mitochondria morphology, dynamics, and function. Our results demonstrate that we can mechanically characterize several thousands of isolated mitochondria within minutes and are able to estimate their Young's modulus as a label-free intrinsic material parameter. Exposing C6 glial cells to varying concentrations of hydrogen peroxide (H_2_O_2_) to induce superoxide as ROS leads to a reduction in mitochondria size and in increased deformation. Interestingly, the knockout of the *tafazzin* gene, known to play a major role in patients suffering from the Barth syndrome (BTHS), leads to the same biomechanical phenotype. Taken together, our study highlights the potential of using mechanical properties as an indicator for mitochondrial (dys-) function.

## MATERIALS AND METHODS

### Cell culture

C6 glial cells (Deutsche Sammlung von Mikroorganismen und Zellkulturen—DSMZ) and the corresponding *tafazzin* knockout model (*Taz^−/−^*) were generated as described previously.^35^ Cells were cultured in DMEM (PAN-Biotech) supplemented with 3% fetal calf serum (FCS, Gibco, ThermoFisher Scientific), 1% penicillin/streptomycin (BioWest), and 2 mM l-glutamine (BioWest) at 37 °C and 5% CO_2_. Cells were passaged every 48 h by washing with PBS (BioWest) and enzymatically detached by incubating with 1% trypsin (BioWest) for 3 min. The reaction was stopped by adding a cell culture medium followed by the collection of cells into a falcon and centrifugation at 200×  RCF for 5 min.

### Mitochondria isolation

Mitochondria isolation was performed according to the protocol modified from Gürtler *et al.*^35^ Approximately, 1 × 10^6^ C6 glial cells were seeded per T175 flask and cultured for 4 days with a medium change on day 2. On the fourth day, cells were washed once with PBS at room temperature, followed by detaching cells by scraping in the presence of ice-cold PBS and centrifugation at 600 × RCF for 5 min at 4 °C. Cells were resuspended in 6 ml of a hypotonic buffer (10 mM NaCl, 1.5 mM MgCl_2_, and 10 mM Tris–HCl) for 4 min, and the cell pellet was collected after centrifugation at 600× RCF for 5 min at 4 °C. The supernatant was removed and the pellet containing swollen cells was resuspended in 1 ml of freshly prepared mitochondria isolation buffer (210 mM mannitol, 70 mM sucrose, 5 mM Tris–HCl, with pH 7.5, 1 mM EDTA, and 0.1% BSA). The cell suspension was passed five times through a 29G, U-100 syringe needle (Brown) to burst open the cells. Another 5 ml of isolation buffer was added to the lysate and transferred into 2 ml vials (Eppendorf, Germany) and centrifuged at 4 °C, 600× RCF for 10 min to separate the lysate from the remaining cells. The supernatant was transferred into fresh tubes and centrifuged at 17 000× RCF for 15 min at 4 °C. The pellet was washed once in the isolation buffer followed by centrifugation at 4 °C and 17 000× RCF for 10 min. The final pellet contained isolated mitochondria, as previously reported.^36^ Adaptation of this protocol would also enable us to extract cell nuclei^37^ and at higher centrifugation speeds (>20 000× RCF), the isolation of other organelles like lysosomes.

### Functional assessment of isolated mitochondria

MitoSPY Green FM (Biolegend) was used as a fluorescent indicator for mitochondria inside living cells. The dye is not based on the membrane potential and, thus, can be used to measure the mitochondrial mass of a cell.^38,39^ The reagent was dissolved in dimethyl sulfoxide (DMSO, Carl Roth). From a stock concentration of 1 mM MitoSPY-green, a dilution of 1:1000 was prepared using mitochondria isolation buffer, added to the mitochondria pellet and incubated for 10 min at 4 °C. After centrifugation at 4 °C and 17 000× RCF for 10 min, the pellet was washed once by resuspending in fresh buffer followed by another centrifugation step at 4 °C and 17 000× RCF for 10 min. As a vehicle control, mitochondria were exposed to DMSO and the same washing procedure was applied.

To perform intracellular imaging of mitochondria, cells were cultured in eight-well chamber slides (ibdi) and incubated for 36 h at 37 °C and 5% CO_2_. After incubation, the culture medium was removed and the cells were washed once with room temperature PBS and stained with MitoSPY-green at 1:200 dilution. In addition, the nucleus was fluorescently labeled by NucBlue^TM^ (ThermoFisher Scientific) following the manufacturer's protocol. After two additional washing steps with PBS, live cell imaging was performed by epi-fluorescence microscopy using an Eclipse Ti-microscope (Nikon) equipped with an iXon + 897 EMCCD camera (Andor), INUB-GSI stage top incubator (Tokai Hit) and a GM-4000 Gas Mixer (Tokai Hit). Images were acquired using a 63× objective and further processed by background subtraction using ImageJ (version 2.3.0/1.53j) to reduce the noise and improve the contrast.^40,41^

### Fluorescent detection of mitochondrial superoxide

MitoSOX^TM^ Red (ThermoFisher Scientific) was used as a fluorescent indicator to analyze the mitochondrial superoxide levels in intact C6 cells and isolated mitochondria. The reagent was dissolved in DMSO (Carl Roth) to prepare a 5 mM stock solution. For detecting intracellular mitochondrial superoxide induced by H_2_O_2_, C6 cells were first incubated with 2.5 *μ*M MitoSox-red in 1 ml PBS for 10 min and washed subsequently with PBS before adding H_2_O_2_ in the respective concentrations. The cells were pelleted by centrifugation at 200 × RCF for 5 min. The cell pellet was resuspended in Cell Carrier-A buffer (CCA, Zellmechanik Dresden), and the fluorescence intensity was measured.

To analyze superoxide levels in isolated mitochondria from C6 wild-type (WT) and *Taz^−/−^* cells, we used a 1:1000 dilution of MitoSOX-red in mitochondria isolation buffer and incubated on ice for 10 min, followed by two washing steps at 4 °C and 17 000× RCF for 10 min each.

### H_2_O_2_ treatment

Hydrogen peroxide (H_2_O_2_) was used to induce mitochondrial superoxide as an intracellular ROS.^42^ From a 10 M H_2_O_2_ stock (Honeywell), a working solution of 100 mM H_2_O_2_ was prepared in PBS, which was further diluted and added to C6 cells at final concentrations of 35 and 1000 *μ*M, respectively, followed by a 30 min incubation at 37 °C and 5% CO_2_. As a control, we used 0 *μ*M H_2_O_2_ treated for the same time and at an equal temperature. After incubation, mitochondria were isolated from cells (see above) for mechanical characterization. For evaluation of intracellular superoxide levels, cells were pretreated with MitoSOX-red (see above) and fluorescence intensity was measured after the respective H_2_O_2_ treatment.

### Real-time fluorescence and deformability cytometry

*Acquisition:* Real-time fluorescence and deformability cytometry (RT-FDC) is a microfluidic technique (AcCellerator, Zellmechanik Dresden), which enables parallel mechanical and fluorescence-based sample characterization of individual cells in suspension at a throughput exceeding 1000 cells per second.^20,21^ The setup of RT-FDC is assembled on an inverted microscope equipped with a fluorescence module and a CMOS camera which captures images at 4000 frames per second. Microfluidic chips are made of polydimethylsiloxane (PDMS) with a central constriction of 300 *μ*m in length and a cross section of 15 × 15 *μ*m (Flic15, Zellmechanik Dresden) for mitochondria and 30 × 30 *μ*m (Flic30, Zellmechanik Dresden) for cells, respectively. Isolated mitochondria were resuspended in a measurement buffer consisting of 1% MC [(w/v), methylcellulose, (Sigma-Aldrich) in PBS^−/−^ (without Ca^2+^ and Mg^2+^)] and analyzed at flow rates of 8, 12, and 16 nl/s using a 63× magnification objective with oil immersion and an optical resolution of 0.22 *μ*m/pixel. Cells resuspended in Cell Carrier-A were analyzed at a flow rate of 160 nl/s using a 40× magnification air immersion objective.

For C6 cells and isolated mitochondria, the mechanical properties such as deformation and the level of mitochondrial superoxide could be measured simultaneously using RT-FDC. The deformation is calculated by the formula:^21^

$$\begin{array}{c} \mathrm{Deformation}=1-\mathrm{Circularity}\;=1-\frac{2\sqrt{\pi \;\mathrm{Area}}}{\mathrm{Perimeter}}, \end{array}$$ (1)

where cell area and perimeter are extracted from the cell contour obtained by a border-following algorithm.^43^ For a perfectly round object, the circularity is 1 and thus the deformation is zero.

For fluorescence analysis, an excitation wavelength of 561 nm and an emission filter of 593/46 nm was used. In addition, mitochondrial viability was confirmed by MitoSPY-green staining (see above) at an excitation wavelength of 488 nm as well as an emission filter of 525/50 nm. In a typical experiment, 20 000 events were measured, using the proprietary software ShapeIn (version 2.0.5, Zellmechanik Dresden).

*Analysis:* Data analysis was performed in Shape-Out (version 1.0.5, Zellmechanik Dresden). For mitochondria, an area-ratio filter of 1.1 was applied to account for a 10% maximum deviation of the convex hull area from the (raw) contour area.^21–45^ The resulting events were gated for a size between 0.3 and 10 *μ*m^2^ to exclude small debris and mitochondrial clusters. In a typical measurement between 3000 and 6000, mitochondria can be analyzed after applying all filters. For whole cells, an area-ratio filter of 1.05 was applied to account for a 5% maximum deviation of the convex hull area from the (raw) contour area.^21^ In addition, a cell size gate between 50 and 600 *μ*m^2^ has been applied.

### Measurement buffer viscosity

The viscosity of the measurement buffer for mitochondria [MC 1% (w/v) diluted in the PBS^−/−^] was measured using a rheometer (MCR502, Anton Paar, Ostfildern, Germany) with cylinder geometry (CC28.7, CC27-SS Anton Paar). The temperature was set to 25 °C and the shear rate ranged from $\dot{\gamma }=1$ to 40 000 1/*s*. Within this range, the viscosity η follows a power law^46,47^

$$\begin{array}{c} \eta =k\cdot {\left(\frac{\dot{\gamma }}{{\dot{\gamma }}_{0}}\right)}^{m-1}, \end{array}$$ (2)

with a power-law exponent *m* = 0.522, a proportionality factor *k* = 1.936 Pa s and ${\dot{\gamma }}_{0}=1/s$ for reference.

### Hydrodynamic simulations

Numerical simulations utilizing the finite element method (FEM) are implemented in COMSOL Multiphysics 6.0 and its CFD module (Comsol Multiphysics GmbH) to estimate the mechanical properties of suspended mitochondria. Using the creeping flow interface, an incompressible flow is modeled in a 2D axisymmetric geometry neglecting inertial contributions and turbulence. The resulting Stokes flow is solved in the central region of a microfluidic channel of 80 *μ*m length and a square channel of 15 × 15 *μ*m cross section, as previously described.^44^

A mitochondrion, modeled first as a sphere and second as a rod consisting of two hemispheres as well as a connecting cylinder, is placed in the center of the channel. The shape of mitochondria was observed to change between spherical and rod during the fission process and when the cell membrane is disrupted.^48^ Also, the shape of mitochondria varied between the wild-type and *tafazzin* knockout of C6 glial cells. Taking from experimental data, the sphere has a diameter of 1.26 *μ*m while the rod is modeled with a diameter of 1.26 *μ*m and a length of 1.6 *μ*m. Simulations are performed for volumetric flow rates of 6, 8, 12, and 16 nl/s, respectively.

At the channel inlet, a fully developed laminar flow with the respective volumetric flow rate is established, while at the outlet, a constant pressure constraint is set and normal flow is enforced. The fluid consists of 1% MC (w/v) characterized by a mass density of 1065 kg m^−3^ (DMA4500, Anton Paar) and a shear-thinning behavior determined by a power law as described above [Eq. (2)]. Viscosities in close proximity to the mitochondrion are in the order of 270 mPa s for experimentally relevant shear rates of approximately 200 1/s (Tables S5 and S6 in the supplementary material). Channel walls and the mitochondrion surface are determined by the no-slip boundary condition. Equilibrium velocities were found to be 47.2, 63.0, 94.5, and 126.0 mm/s for the four flow rates, respectively, by balancing the normal and shear forces on the mitochondrion surface exerted by the surrounding fluid.

### Statistical analysis

Statistical analysis was performed using a linear mixed model approach on data obtained from three biological replicates using Shape-Out (version 1.0.5, Zellmechanik Dresden). A pairwise comparison was done between the two groups and the differences in an observable property, e.g., the deformation, attributed to random and fixed effects, respectively. Random effects account for the difference in concentrations of fluorescent dye or variations in background illumination (RT-FDC) between the replicates and the fixed effects represent the actual effect size, i.e., fold change of an experimental quantity. Statistical significance was analyzed using two models, one with and one without the fixed effects, and the maximum likelihoods are calculated. From the likelihood ratio and applying Wilks' theorem, the resultant *p*-values are determined.^45^ The data obtained were plotted using GraphPad Prism 7 (version: 7.0e, GraphPad Software). The results of the mechanical characterization of isolated mitochondria are expressed as the mean ± standard error of the mean (SEM).

## RESULTS

### Optomechanical characterization of isolated mitochondria in flow

**FIG. 1.**
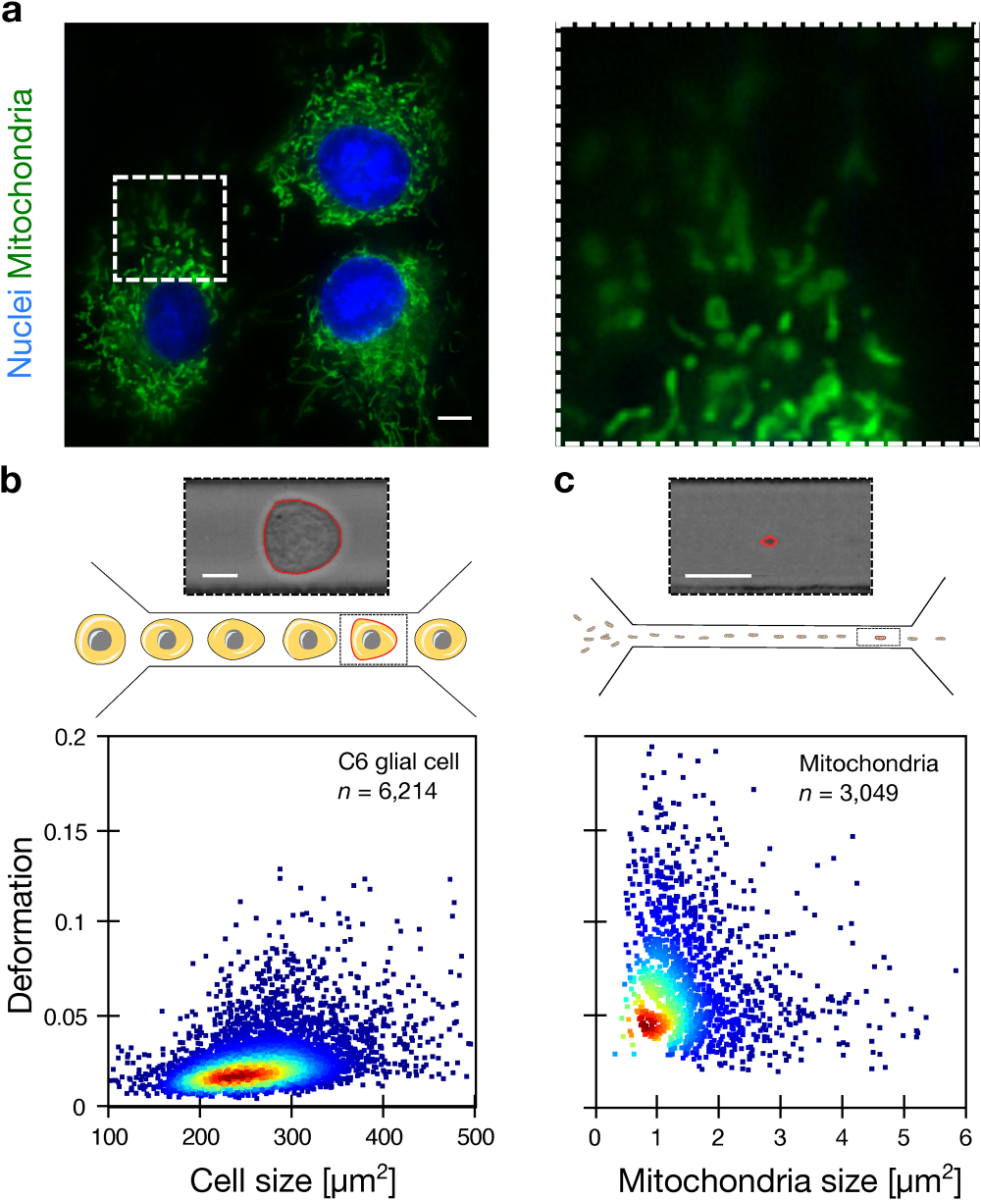
Cell and mitochondria optomechanics. (a) Live cell imaging of C6 glial cells labeled with NucBlue (nucleus, blue) and MitoSPY-green (mitochondria, green). The inset is highlighted on the right (dashed line). (b) Schematics of the microfluidic channel of 30 × 30 *μ*m^2^ cross section and microscopic image of a deformed cell at channel outlet (top). The scatter plot shows the deformation vs size of C6 glial cells measured by RT-FDC at a flow rate of 160 nl/s (bottom). (c) Schematics of the microfluidic channel of 15 × 15 *μ*m^2^ cross section and microscopic image of a deformed mitochondrion at the channel outlet (top). The scatter plot shows the deformation vs size of isolated mitochondria measured by RT-FDC at a flow rate of 8 nl/s (bottom). The red solid line around the cell and mitochondrion in (b) and (c) corresponds to the contour and is used to calculate size and deformation. Scale bars 10 *μ*m.

Prior to mechanical characterization, fluorescent imaging exemplified an intracellular organization of individual as well as fused networks of mitochondria, which are perinuclearly distributed inside cells [Fig. 1(a)]. Real-time fluorescence and deformability cytometry (see Methods, RT-FDC) on C6 cells and isolated mitochondria were performed in microfluidic channels of 30 × 30 and 15 × 15 *μ*m^2^ cross section, respectively.^20^ Within the constriction, cells usually adapt a bullet-like shape [Fig. 1(b), sketch and brightfield image], while mitochondria are elongated as expected [Fig. 1(c), sketch and brightfield image]. The optomechanical analysis of *n* = 6214 glial cells and *n* = 3049 mitochondria yielded homogeneous populations with an average size of 346 ± 103.2 *μ*m^2^ [median ± standard deviation (SD), Fig. 1(b), lower panel] for cells and 1.01 ± 0.88 *μ*m^2^ [Fig. 1(c), lower panel] for mitochondria. The deformation of cells was lower (0.044 ± 0.026) compared to mitochondria (0.057 ± 0.036).

### Deformation of isolated mitochondria is shear stress-dependent

**FIG. 2.**
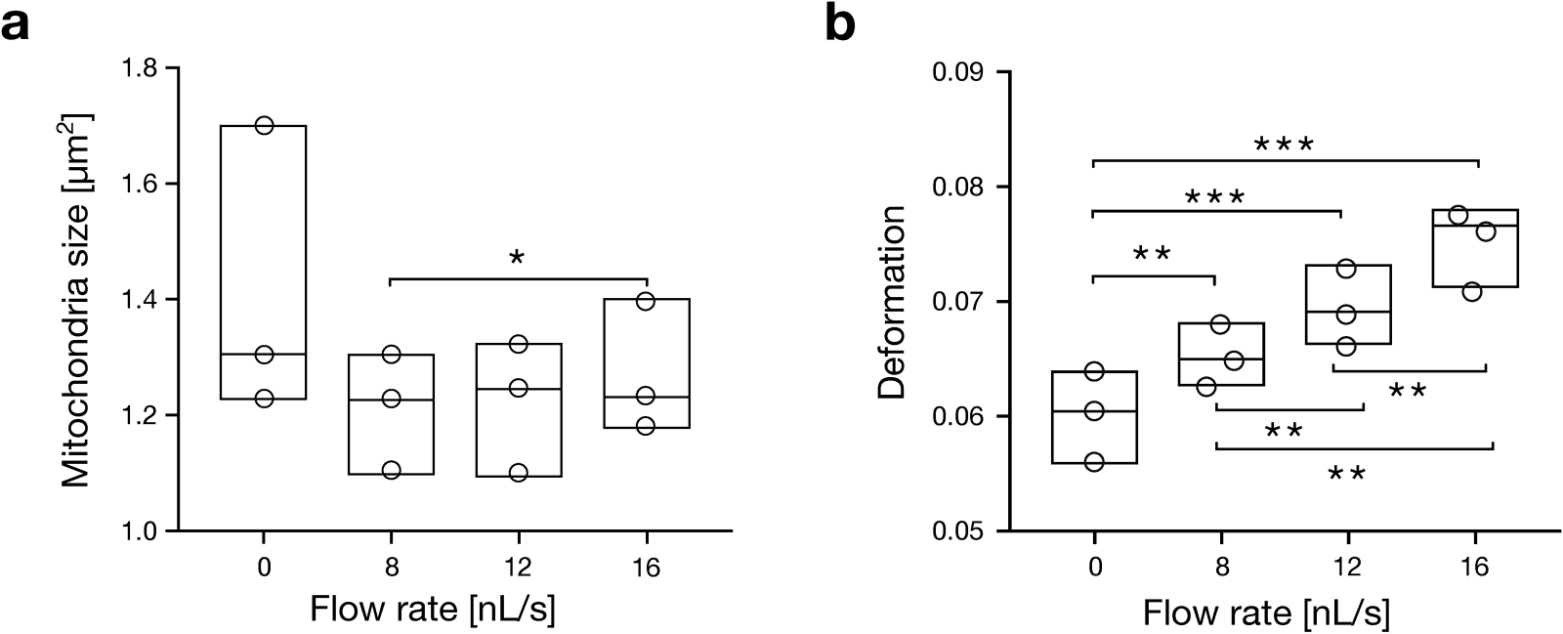
Flow rate dependency of size and deformation. Statistical analysis of (a) mitochondria size and (b) mitochondrial deformation at different flow rates (8, 12, and 16 nl/s) compared to stress-free condition (0 nl/s). Data summarize triplicates with *n* = 5059 for 0 nl/s, *n* = 10 061 for 8 nl/s, *n* = 10 207 for 12 nl/s, *n* = 10 019 for 16 nl/s single mechanical measurements on isolated mitochondria. Statistical analysis was done using linear mixed models (*, p < 0.05, **, p < 0.01, ***, p < 0.001).

The small size of mitochondria (∼1 *μ*m) poses specific challenges in determining their mechanical properties as optical microscopy only provides a finite number of pixels for image analysis. First, the viability of isolated mitochondria has been verified by flow cytometry using MitoSPY-green (see Methods). Here, around 99% of mitochondria measured were found functionally active (Fig. S1 and Table S1 in the supplementary material). For answering the question if mitochondria can be deformed using a microfluidic assay like RT-FDC, we applied flow rates of 8, 12, and 16 nl/s and compared the results with control measurements in a reservoir where hydrodynamic stresses can be neglected. The statistical analysis (see Methods) of experimental triplicates consisting of a total of 35 346 isolated mitochondria revealed a mean size of 1.29 ± 0.05 *μ*m^2^ (mean ± standard error of the mean, SEM) in the absence of stress, 1.24 ± 0.06 *μ*m^2^ at a flow rate of 8 nl/s and 1.28 ± 0.05 *μ*m^2^ at a flow rate 16 nl/s respectively [Fig. 2(a), Figs. S2 and S3 and Table S2 in the supplementary material]. While mitochondrial size revealed little flow rate dependency, deformation shows a positive correlation. At the lowest flow rate (8 nl/s), deformation was significantly elevated (0.070 ± 0.003) compared to the stress-free condition (0.060 ± 0.001) and increased further to 0.074 ± 0.003 at the highest flow rate (16 nl/s). Significant changes were observed between all conditions [Fig. 2(b), Figs. S2, S3 and Table S2 in the supplementary material].

The small size of mitochondria and their relatively high velocity of approximately 10 cm/s inside our microfluidic system lead to motion blur that could artificially increase the observed deformation values. We approximated the motion blur (%) by

$$\begin{array}{c} \mathrm{Motion}\;\mathrm{blur}\;(\text{\%})=\frac{v\times t_{\mathrm{shutter}}\;}{D}\times 100,\; \end{array}$$ (3)

with *v* corresponding to the experimental velocity of mitochondria at a given flow rate inside a 15 × 15 *μ*m^2^ channel, $t_{\mathrm{shutter}}$ is the camera shutter time of 2.4 *μ*s, and *D* is the mitochondrial diameter. We calculated a motion blur of 14% at a flow rate of 8 nl/s, which increased to 19% at 12 nl/s and to 27% at 16 nl/s (Table S3 in the supplementary material). At 12 nl/s, the amplitude of motion blur compares to a cell measurement inside a 30 × 30 *μ*m^2^ channel at a flow rate of 320 nl/s (17%), which is routinely used in RT-DC experiments. For the present work, we aim to minimize the role of motion blur in deformation measurements and opted for the flow rate of 8 nl/s to carry out all further experiments.

### Estimation of mitochondrial elasticity

Next, we aimed to determine the Young's modulus of isolated mitochondria. Since their small size leads to potential pixelation artifacts^49^ and renders the application of the analytical and numerical models^44–50^ challenging, we decided to estimate the elasticity from the hydrodynamic stress distribution on the mitochondrial membrane. Here, we assumed that the lowest flow rate of 8 nl/s, where we still observe a finite deformation, corresponds to an amplitude in hydrodynamic stress that imposes an upper limit in Young's modulus of isolated mitochondria.

Since our mitochondrion-channel system can be described by a laminar flow, the hydrodynamic stress σ can (in a first approximation) be calculated from Newton's law of viscosity

$$\begin{array}{c} \sigma =\eta \cdot \dot{\gamma }, \end{array}$$ (4)

where η is the viscosity of the carrier buffer (MC 1%) and $\dot{\gamma }$ is the shear rate inside the microfluidic channel^44^

$$\;\dot{\gamma }=\frac{v}{R_{c}-R_{m}}.$$ (5)

Here, $R_{c}=7.5\;\mu m$ is the radius of the constriction [Fig. 1(c), sketch] and $R_{m}=0.63\;\mu m$ is the radius of mitochondria, which is approximated from their bounding box. Please note, being a shear-thinning solution, the viscosity of our carrier buffer is shear rate dependent by itself, i.e., $\eta =\eta (\dot{\gamma })$ and has to be calculated from Eq. (2).

Utilizing typical experimental parameters for a flow rate of 8 nl/s and a corresponding velocity of 7 cm/s, we obtain a shear rate of approximately $\;\dot{\gamma }=10^{4}\;1/s$, a viscosity η=23.5mPas and stress on the mitochondria surface of σ=240Pa from Eqs. (4) and (5) (Table S4 in the supplementary material). Performing the same calculations at a flow rate of 6 nl/s where no finite deformations are observed yields σ=200Pa and allows for estimating Young's modulus $E_{m}$ of isolated mitochondria to $200<E_{m}\leq 240\;\mathrm{Pa}$.

**FIG. 3.**
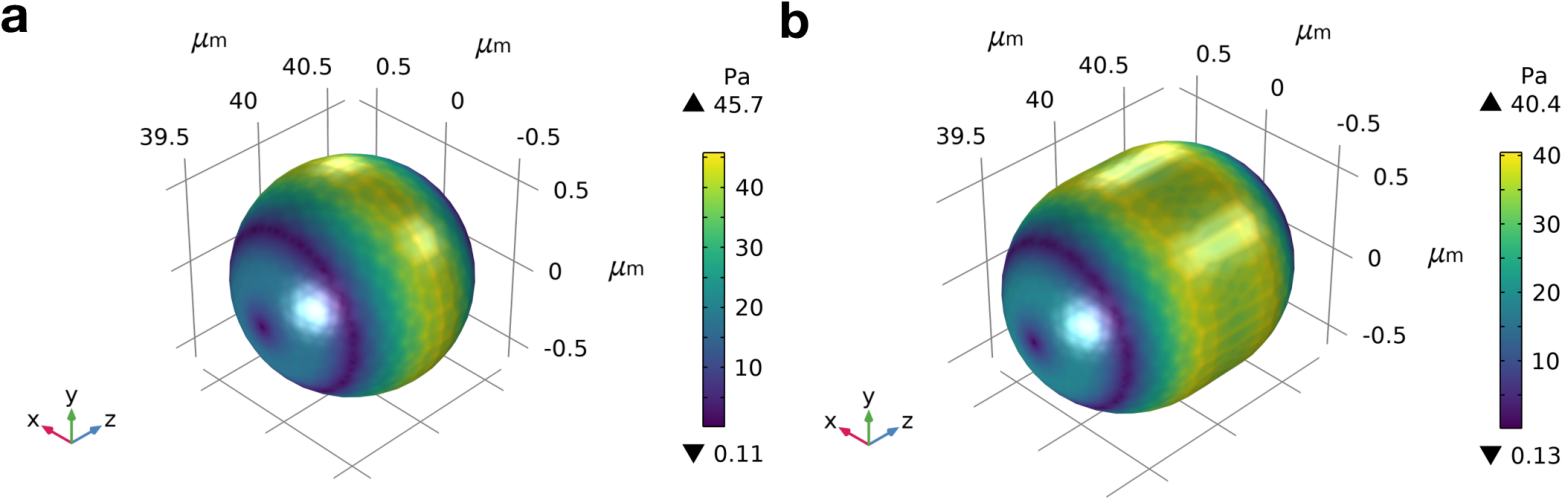
Hydrodynamic stress on mitochondria surface. Finite element method simulations showing the stress exerted by the surrounding fluid on a spherical mitochondrion with a diameter of 1.26 *μ*m (a) and a rod-shaped mitochondrion with a diameter of 1.26 *μ*m and length of 1.6 *μ*m (b). Simulations assumed a channel of 80 *μ*m in length with fully developed laminar flow and a flow rate of 8 nl/s.

Equation (4) is strictly only valid for surfaces close to the walls of the microfluidic channel.^44^ Since the small relative size of mitochondria inside a 15 *μ*m constriction renders this assumption nearly invalid, we also carried out finite element method (FEM) simulations to determine the hydrodynamic surface stress distribution under experimental conditions (see Methods). Simulations were performed for mitochondria in a laminar flow of 8 nl/s (Fig. S4 in the supplementary material) where a peak surface shear stress of approximately 46 Pa was found for spheres [Fig. 3(a) and Table S5 in the supplementary material] and 40 Pa for rods [Fig. 3(b) and Table S6 in the supplementary material]. Repeating calculations for a flow rate of 6 nl/s, where no finite deformation was observed in experiments, yields σ≈40Pa (sphere) as well as σ≈35Pa (rod) and enables us to estimate the elasticity of a single mitochondrion to $35<E_{m}\leq 45\;\mathrm{Pa}$ represented by spheres as well as rods [Figs. 3(a) and 3(b)].

### Increased superoxide levels lead to softening of mitochondria

**FIG. 4.**
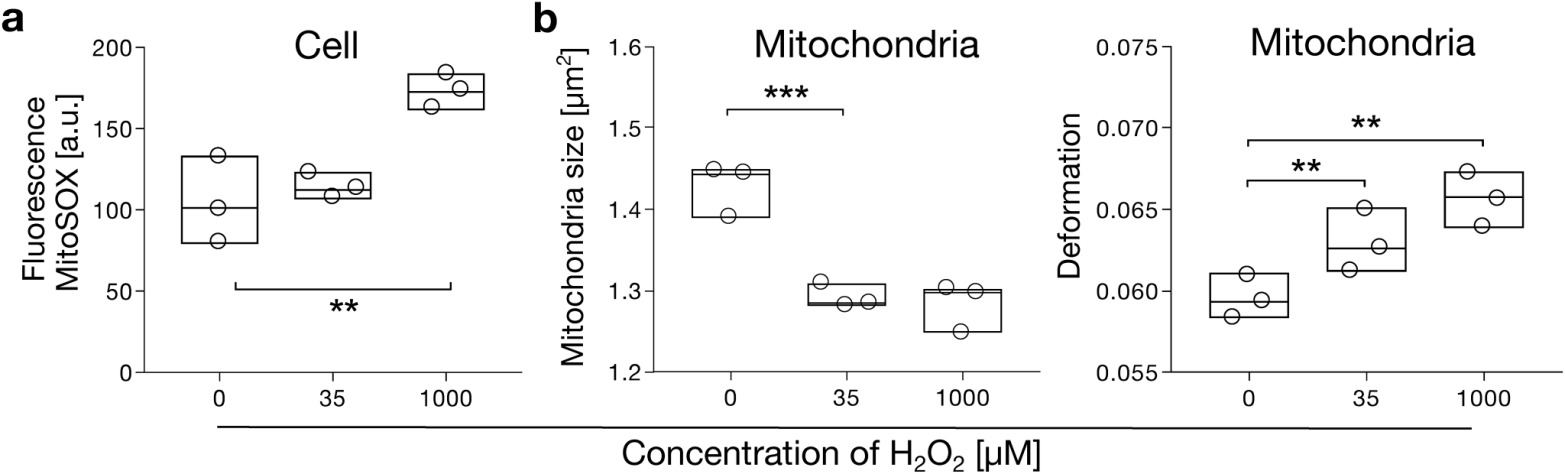
H_2_O_2_ induces mechanical alterations in mitochondria. (a) Statistical analysis of three biological replicates comparing fluorescence intensities of MitoSOX-red stained C6 glial cells at varying H_2_O_2_ concentrations. Analysis summarizes *n* = 11 346 (0 *μ*M H_2_O_2_), *n* = 11 402 (35 *μ*M H_2_O_2_), and *n* = 15 939 (1000 *μ*M H_2_O_2_) single cells. Statistical analysis of (b) size and deformation of isolated mitochondria for experimental triplicates at varying H_2_O_2_ concentrations. Analysis summarizes *n* = 12 314 (0 *μ*M H_2_O_2_), *n* = 10 205 (35 *μ*M H_2_O_2_), and *n* = 9093 (1000 *μ*M H_2_O_2_) individual mitochondria. Measurements have been performed using RT-FDC at a flow rate of 160 nl/s (cells) and 8 nl/s (mitochondria). Analysis was done using linear mixed models (**, p < 0.01, ***, p < 0.001).

Mitochondria are considered a major intracellular ROS producer.^2^ Under normal physiological conditions, ROS homeostasis is maintained by several enzymes such as superoxide dismutase and catalase, but conditions such as aging, cancer, and degenerative diseases are known to be caused by mitochondrial defects and are associated with elevated ROS levels.^3–52^ Here, we explored if the mechanical properties of mitochondria are linked to levels of intracellular superoxide. Answering this question might be of fundamental relevance since shape and curvature play a pivotal role in mitochondrial fission and fusion.^33,34^ In the first set of experiments, C6 glial cells were treated with H_2_O_2_ at different concentrations (0, 35, and 1000 *μ*M). After 30 min of incubation, the presence of intracellular superoxide was verified by MitoSOX-red using flow cytometry (see Methods). While experimental triplicates of a total of *n* = 38 687 cells show nearly no change at 35 *μ*M, fluorescence intensity significantly increased at 1000 *μ*M [Fig. 4(a), Fig. S5 and Table S7 in the supplementary material].

In the next step, we isolated mitochondria from C6 glial cells after H_2_O_2_ treatment (see Methods). Analysis by RT-DC revealed changes in size and deformation for all concentrations of hydrogen peroxide (Fig. S6 in the supplementary material), indicating mitochondrial alterations due to increased oxidative stress. Data of experimental triplicates of *n* = 31 612 isolated mitochondria demonstrate a significant decrease in size from 1.43 ± 0.08 *μ*m^2^ at 0 *μ*M H_2_O_2_ to 1.29 ± 0.3 *μ*m^2^ at 35 *μ*M H_2_O_2_. At 1000 *μ*M, the size remains nearly constant at 1.28 ± 0.03 *μ*m^2^ [Fig. 4(b), left panel]. In contrast, the deformation of isolated mitochondria significantly increases with increasing H_2_O_2_ concentrations from 0.060 ± 0.001 at 0 *μ*M H_2_O_2_ to 0.063 ± 0.001 at 35 *μ*M H_2_O_2_ and 0.066 ± 0.001 at 1000 *μ*M H_2_O_2_ [Fig. 4(b), right panel, Fig. S7 and Table S8 in the supplementary material].

### Knockout of *tafazzin* increases mitochondrial deformation

**FIG. 5.**
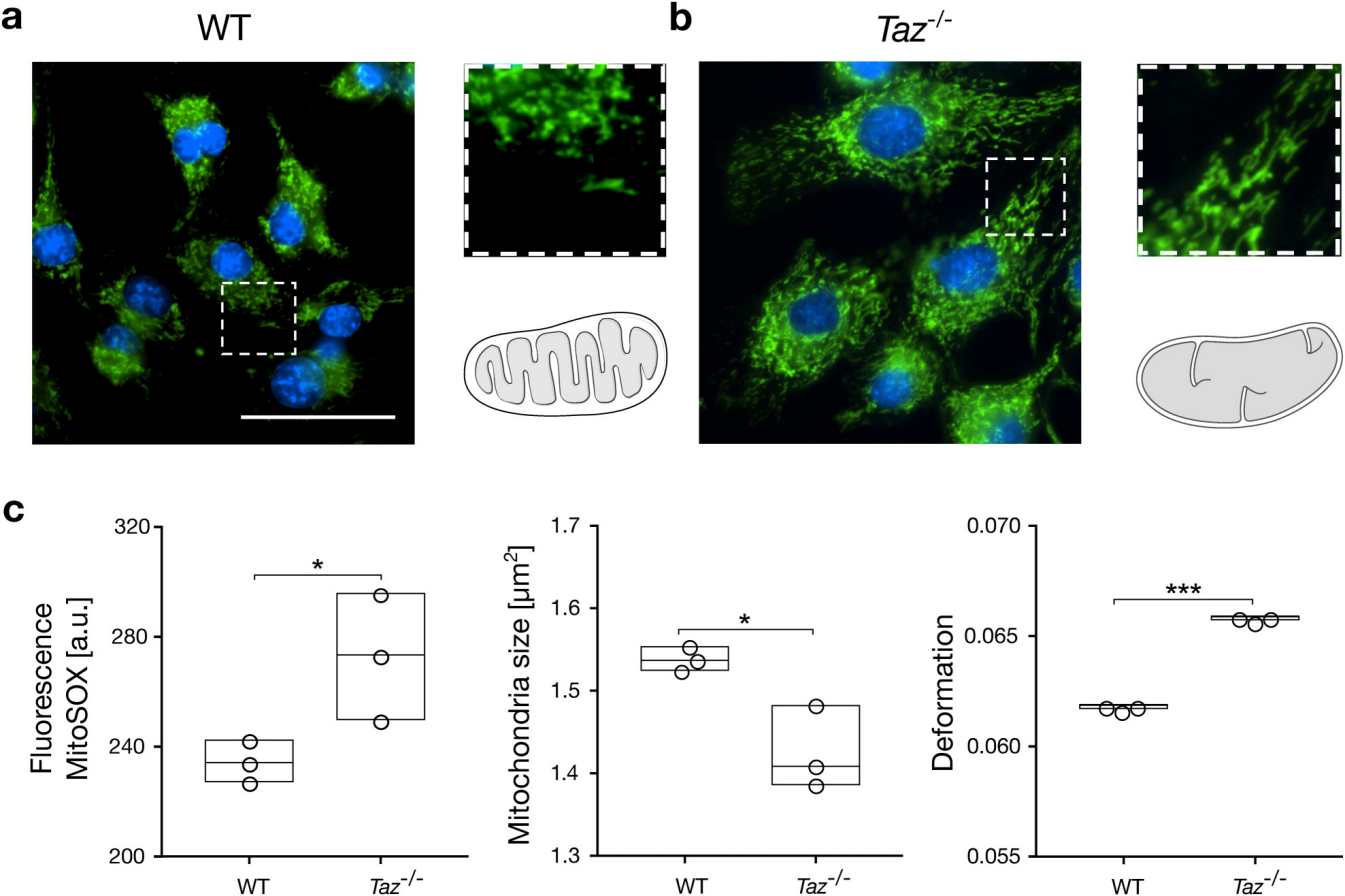
Knockout of *tafazzin* alters mitochondria size and deformation. Fluorescence images of mitochondria (green) and nucleus (blue) inside (a) WT and (b) *Taz^−/−^* C6 glial cells. The inset shows the magnified region (dashed line) and the sketch illustrates the change in inner mitochondria membrane shape between WT and *Taz*^−/−^ cells. The scale bar is 50 *μ*m. (c) Statistical analysis of superoxide levels (left panel), mitochondria size (center panel), and mitochondria deformation (right panel) for three biological replicates of mitochondria isolated from WT and *Taz*^−/−^ cells. Data summarize a total of *n* = 8942 mitochondria for WT conditions and *n* = 7153 mitochondria for *Taz^−/−^* conditions. Measurements have been performed at a flow rate of 8 nl/s. Statistical analysis was performed using linear mixed models (*, p < 0.05; ***, p < 0.001).

Finally, we were interested to investigate the effect of increased levels of intracellular ROS on mitochondria mimicking a disease state. To investigate this, we chose a *tafazzin* gene knockout model of C6 glial cells (*Taz*^−/−^), which was shown to lead to higher mitochondrial superoxide levels.^35^ *Tafazzin* is an essential enzyme of cardiolipin remodeling and for the proper assembly of respiratory chain (super-) complexes.^53–55^ For qualitative analysis, mitochondria of wild-type (WT) and *tafazzin* knockout cells were visualized by fluorescent microscopy after labeling with MitoSPY and NucBlue (see Methods). We observed differences in mitochondrial structure and organization with WT cells [Fig. 5(a), top right and Fig. S8 in the supplementary material] possessing shorter mitochondria compared to the *Taz*^−/−^ cells [Fig. 5(b) top left and Fig. S9 in the supplementary material]. The schematic of both WT [Fig. 5(a), bottom right] and *Taz^−/−^* [Fig. 5(b), bottom right] mitochondria illustrated the change in the inner mitochondrial membrane, especially the disorganized cristae formation as reported earlier.^56,57^

In our quantitative analysis, we studied experimental triplicates of a total of 16 095 mitochondria. Before focusing on the mechanical properties, we first verified elevated levels of superoxide inside mitochondria isolated from *Taz^−/−^* cells using MitoSOX-red [Fig. 5(c), left panel, Fig. S10 and Table S9 in the supplementary material]. The size of isolated mitochondria decreased significantly between WT (1.54 ± 0.01 *μ*m^2^) and *Taz^−/−^* [1.43 ± 0.04 *μ*m^2^; Fig. 5(c), center panel], while we found a significantly increased deformation from 0.062 ± 0.001 for WT to 0.066 ± 0.001 for *Taz*^−/−^ [Fig. 5(c), right panel, Figs. S11, S12 and Table S9 in the supplementary material]. Interestingly, the biophysical response of mitochondria to elevated ROS levels was independent of the way it has been induced, i.e., exogenously by incubation with H_2_O_2_ or endogenously by *tafazzin* knockout.

## DISCUSSION

Mitochondria are essential organelles for maintaining cellular homeostasis by controlling energy synthesis, cellular metabolism, and the formation of ROS.^2–59^ Analysis of mitochondrial mechanics and other biophysical properties like morphology, area, aspect ratio, and volume can reveal the cellular state and have already been used as a biomarker for organelle function under physiological as well as pathological conditions.^7^ Relevant methods include force spectroscopy by AFM, laser tweezers Raman spectroscopy (LTRS), and micropipette aspiration.^7–60^ AFM analysis of the morphological properties of normal and ischemic rat heart mitochondria revealed that myocardial infarction might cause mitochondrial swelling and changes in the adhesion force and stiffness.^7^ Physio-chemical changes in the cellular environment also showed effects on mitochondrial dynamics. Mitochondrial deformation and stiffness were significantly affected by changes in osmotic pressure, pH, and calcium levels when analyzed using micropipette aspiration and LTRS.^31–60^ However, these methods are, in general, limited by throughput, which is relevant when a sufficient sample size is required while simultaneously ensuring mitochondrial viability.

Here, we introduced RT-FDC for a high-throughput and label-free characterization of mitochondria mechanics in real time.^20^ Besides the throughput and capability to analyze mitochondria in suspension, our approach also enables simultaneous fluorescence detection, e.g., to assess the viability of each individual organelle as well as the amount of superoxide. We use our method to demonstrate that isolated mitochondria from rat C6 glial cells form a homogeneous population in size and deformation. Aiming to derive material properties, i.e., Young's modulus, we follow an analytical and numerical approach. Estimation of mitochondria elasticity has been done before using T-Rex-293 kidney cells and performing micropipette aspiration studies.^31^ The results by Wang *et al.* indicated organelle deformation at suction pressures as low as 25 mm H_2_O corresponding to an area compressibility modulus of 9 mN/m. Using force spectroscopy by AFM, Lee and co-workers estimated the membrane tension of mitochondria inside fixed rat heart cells to 0.2 nN/nm.^7^ In contrast, Janel *et al.* report an apparent elasticity of up to 100 kPa for mitochondria isolated from PtK2 kidney epithelial cells.^61^

While AFM and micropipette aspiration impose a local stress or line stress, respectively, RT-FDC makes use of a hydrodynamic flow field that leads to shear and normal stress on all mitochondrial surfaces. This might explain why our numerical analysis yields a Young modulus between 35 and 45 Pa, which is approximately a factor of 1000 lower than the current state of the art. Besides the differences in methodology, the time scale of measurement, and data analysis, our results could also be explained by a cell type specificity pointing to the question of to what extent material properties of mitochondria are conserved across different tissues.

In a first proof-of-principle study, we applied our method to explore the applicability of mechanical properties as a label-free marker for ROS-induced mitochondria alterations. Increased ROS levels are believed to be common observations in diseases such as mitochondrial myopathy, cancer, Parkinson's, diabetes, ischemia, and also during aging.^59–63^ We simulated a disease environment *in vitro*, by systematically triggering ROS-driven stress response by treating C6 cells with H_2_O_2_ in the micromolar and millimolar range, respectively.^64^ Our data not only show an increased mitochondrial superoxide level with increasing H_2_O_2_ concentrations, as expected, but also a reduction in size and an increase in deformation. The corresponding reduction in mitochondria stiffness might provide a biophysical explanation of mitochondria dynamics under pathological conditions. It has been reported before that increased ROS levels can promote higher fission leading to mitochondrial fragmentation,^9–48^ which could be very well explained by altered membrane tension impacting mitochondria mechanics.

Finally, we studied the Barth syndrome (BTHS) as a disease model, which is an X-linked genetic disorder leading to mitochondrial defects.^57–65^ The main cause of BTHS is a mutation in the *tafazzin* gene, an acyltransferase with a key function in cardiolipin remodeling as well as maturation and, thus, assembly of respiratory chain complexes.^54–65^ Tafazzin deficiency has been associated with a reduced mitochondrial oxidative phosphorylation system, leading to dilated cardiomyopathy and skeletal muscle weakness typically observed in BTHS patients.^56^ In our work, we analyzed isolated mitochondria from the *tafazzin* knockout model (*Taz^−/−^*) of C6 glial cells^35^ and respective wild-type (WT) cells. Our results demonstrate elevated levels of superoxide in *Taz^−/−^* accompanied by a reduction in size and an increase in deformation compared to WT. Our observations could be explained by a loss of membrane stability due to *Taz*^−/−^, which has been reported before.^35–66^

## CONCLUSIONS

We demonstrated that the mechanical properties of isolated mitochondria can be characterized at high throughput using real-time fluorescence and deformability cytometry. The throughput of our microfluidic system enables the recording of large sample sizes and a statistically robust analysis within a short time. This is important for experimental assays since the lifetime of isolated mitochondria is usually limited to approximately 4 h.^67^ In two proof-of-principle studies, our work highlights that exogenously and endogenously produced ROS impacts similarly on the size and mechanical properties of mitochondria with potential relevance for their function. In the future, our assay could be expanded to other organelles, e.g., the cell nucleus, to investigate how alterations in its molecular state and biochemical environment impact nuclear mechanics. We strongly believe that our results pave the way towards the label-free analysis of organelles under physiological and pathological conditions while providing insights into intrinsic material features in a robust statistical fashion that would be hardly accessible by alternative approaches.

## SUPPLEMENTARY MATERIALS

See the supplementary material for supporting graphs, simulations, and tables.

## Data Availability

The data that support the findings of this study are accessible from the corresponding author upon reasonable request.
